# Hepatitis E Virus: Foodborne, Waterborne and Zoonotic Transmission

**DOI:** 10.3390/ijerph10104507

**Published:** 2013-09-25

**Authors:** Danielle M. Yugo, Xiang-Jin Meng

**Affiliations:** Department of Biomedical Sciences and Pathobiology, College of Veterinary Medicine, Virginia Polytechnic Institute and State University, 1981 Kraft Drive, Blacksburg, VA 24061, USA.; E-Mail: mydan12@vt.edu

**Keywords:** hepatitis E virus, HEV, zoonosis, animal reservoir, foodborne transmission, zoonotic transmission, waterborne transmission

## Abstract

Hepatitis E virus (HEV) is responsible for epidemics and endemics of acute hepatitis in humans, mainly through waterborne, foodborne, and zoonotic transmission routes. HEV is a single-stranded, positive-sense RNA virus classified in the family *Hepeviridae* and encompasses four known Genotypes (1–4), at least two new putative genotypes of mammalian HEV, and one floating genus of avian HEV. Genotypes 1 and 2 HEVs only affect humans, while Genotypes 3 and 4 are zoonotic and responsible for sporadic and autochthonous infections in both humans and several other animal species worldwide. HEV has an ever-expanding host range and has been identified in numerous animal species. Swine serve as a reservoir species for HEV transmission to humans; however, it is likely that other animal species may also act as reservoirs. HEV poses an important public health concern with cases of the disease definitively linked to handling of infected pigs, consumption of raw and undercooked animal meats, and animal manure contamination of drinking or irrigation water. Infectious HEV has been identified in numerous sources of concern including animal feces, sewage water, inadequately-treated water, contaminated shellfish and produce, as well as animal meats. Many aspects of HEV pathogenesis, replication, and immunological responses remain unknown, as HEV is an extremely understudied but important human pathogen. This article reviews the current understanding of HEV transmission routes with emphasis on food and environmental sources and the prevalence of HEV in animal species with zoonotic potential in humans.

## 1. Introduction

Hepatitis E virus (HEV), the causative agent of hepatitis E in humans, is an important public health disease in many parts of the World [1,2,3,4]. Transmission is primarily via the fecal-oral route through contaminated food or water [5]. In developing countries in Asia and Africa, poor sanitation conditions lead to outbreaks of acute hepatitis E; however, sporadic and autochthonous cases of hepatitis E also occur throughout many industrialized countries in Europe, Asia, and North America [6,7]. In humans, the mortality rate ranges from 0.5–4% for immunocompetent individuals, however, mortality in HEV-infected pregnant women can reach up to 20% and immunocompromised individuals may develop a chronic HEV infection [8,9]. In addition to humans, HEV has been identified in numerous other animal species including wild and domestic swine, deer, chicken, mongoose, rat, ferret, fish, and rabbits with an ever-expanding host range [1,7,10]. Hepatitis E is now a recognized zoonotic disease with swine and likely other animals serving as the reservoir for human infections [1,8]. Food safety associated with HEV contamination is an important public health concern with the recent identification of infectious HEV in meat and meat products and resultant sporadic cases of foodborne hepatitis E in the human population [3,11,12,13,14]. This review article discusses the public and environmental health concerns and risks associated with HEV infection with an emphasis on foodborne and zoonotic transmissions.

## 2. HEV Classification and Biology

### 2.1. Classification

HEV belongs to the genus *Hepevirus* in the family *Hepeviridae* and consists of four recognized genotypes and at least two putative new genotypes [5]. Genotype 1 causes large outbreaks of acute hepatitis E in humans in Asia. Genotype 2 causes outbreaks in humans and includes one Mexican strain and several African strains. Genotype 3 is associated with sporadic, cluster, and chronic cases of hepatitis E in humans, mostly in industrialized countries. Genotype 3 HEV is known to be zoonotic and has also been isolated from domestic and wild swine, deer, mongoose, rats, and rabbits [12,15,16,17,18,19]. Genotype 4 HEV is also zoonotic and is associated with sporadic cases of hepatitis E in humans and infects wild and domestic swine and reportedly cattle and sheep [1,5].

Avian HEV from chickens only shares approximately 50% nucleotide sequence identity with mammalian HEV; therefore, avian HEV likely represents a separate genus [20]. The genus *Avihepevirus* has recently been proposed to include all three known genotypes of avian HEV in chickens (Genotype 1 in Australia and Korea, Genotype 2 in the United States, and Genotype 3 in Europe and China) [1,21,22]. The recently-identified rat HEV shares approximately 59.9% and 49.9% sequence identities with human and avian HEV, respectively, while the ferret HEV shares the highest sequence identity with rat HEV at 72.3% [18,23]. The genus *Orthohepevirus*has has recently been proposed to encompass both the rat and ferret strains of HEV as well as a novel wild boar HEV strain recovered in Japan that differed from the known Genotypes 1–4 HEV isolates by 22.6–27.7% in nucleotide sequence identity [1,24]. A bat HEV was recently identified from African, Central American, and European bats, and due to high sequence diversification from known HEV isolates at 47% amino acid sequence identity, the bat HEV forms a novel phylogenetic clade [25]. The genus *Chiropteranhepevirus* has been proposed to include all variants of the bat HEV [1]. Finally, a strain of HEV was also identified in cutthroat trout in the United States with only 13–27% sequence homology with mammalian or avian hepeviruses leading to a proposal of another tentative genus, *Piscihepevirus*, within the *Hepeviridae* family [1,26]. The nomenclature of HEV will need to be modified in the near future as more genetically-divergent animal strains of HEV are identified.

### 2.2. HEV Biology

The genome of HEV is a single-stranded, positive-sense, RNA molecule of approximately 7.2 kb in size [3,4,27]. The genome consists of three open reading frames (ORFs), a 5′ non-coding region (NCR), and a 3′ NCR [10]. ORF1 encodes non-structural proteins with conserved domains functioning as a methyltransferase, helicase, RNA-dependent RNA polymerase (RdRp), and a papain-like cysteine protease [20,28]. In addition, a hypervariable region (HVR) within ORF1 may play a role in viral pathogenesis despite being shown to have no influence on viral infectivity [29]. ORF2 encodes the immunogenic capsid protein, which interacts with 3′ viral genomic RNA for encapsidation and contains an endoplasmic reticulum signal peptide and 3′ N-glycosylation sites [30,31]. ORF3 encodes a small phosphoprotein with incompletely understood functions; however, the association with cytoskeleton and its necessity for *in vivo* viral infection in rhesus macaques suggests that ORF3 plays a role in viral replication and assembly [20,32,33].

Avian HEV is genetically related to mammalian HEV with conserved genomic organization and function despite a 600 bp sequence deletion [34,35,36]. The capsid protein of avian HEV contains both unique and conserved antigenic epitopes in comparison to the human and swine HEV capsid proteins [37].

The HEV replication cycle is currently not well understood due to a lack of an efficient cell culture system [38]. Heparin sulfate proteoglycans (HSPGs) likely act as receptors for the attachment of the viral capsid protein, and the heat shock cognate protein 70 may be involved in HEV entry into the cell [38]. Following uncoating in the cell, the HEV genomic RNA is likely utilized to translate the non-structural proteins and the viral RdRp is used to produce progeny virus [38]. Both ORF2 and ORF3 are translated from a bicistronic subgenomic RNA [32,39]. The negative-sense HEV RNA indicative of virus replication is detectable in hepatic and extrahepatic tissues of experimentally-infected rhesus macaques and swine [38,40]. Post-translational processing of proteins and mechanisms of virus assembly and release have yet to be fully elucidated, and the viral-host interactions leading to a disease state are also poorly understood [5,20,38]. Development of a robust cell culture system to efficiently propagate HEV in the future should be a priority, and will facilitate our understanding the biology of this important virus.

## 3. HEV Pathogenesis

### 3.1. HEV Infection in Humans

In humans, HEV causes an acute icteric disease that varies in symptoms from subclinical to fulminant hepatitis [4]. The asymptomatic patient typically clears the virus rapidly, while the symptomatic patient experiences clinical signs including anorexia, hepatomegaly, myalgia, jaundice and sometimes abdominal discomfort, nausea, vomiting, and fever [5,41]. In immunocompromised patients such as organ transplant recipients, lymphoma and leukemia patients, or patients with HIV infection, the course of disease may progress to a chronic state with cirrhosis of the liver and persistence of viral shedding [42,43,44,45,46]. Of particular concern is the ability for HEV-infected immunocompromised individuals to develop clinical disease well after the initial exposure [44,45,46,47]. Currently, chronic HEV infection in immunocompromised individuals is an emerging and significant clinical problem. Future studies are warranted to identify the immunological correlates and host factors leading to chronicity.

The typical infection begins with an incubation period of 2 weeks to 2 months and a transient viremia followed by viral shedding in the feces, disappearance of viremia with the onset of clinical signs, and regression of viral shedding with potential jaundice setting in around 2–3 weeks into the infection [46]. The severity of HEV infection is considered dose-dependent and host factors such as concurrent hepatic disease or alcohol overuse may also contribute to the disease course [41]. In studies from France, Germany, the United Kingdom, and the United States, middle-aged, elderly men were more likely to experience autochthonous HEV infection; however, the underlying host factors have not been understood [48,49,50,51]. Of major concern is the relationship between pregnancy and increased mortality rates up to 20% in HEV endemic regions; however, this relationship appears to be geographically dependent and may be associated with other underlying factors such as virus genotype or concurrent infections with other pathogens [4,20,52,53,54]. Complications with concurrent HEV infection during pregnancy include death of both the mother and fetus, abortion, premature birth, and death of the baby shortly after birth [55]. Vertical transmission from the mother to fetus was reported in 33% of cases and HEV RNA was reportedly detected in human colostrum as well [56,57]. Unfortunately it is not understood why pregnancy resulted in severe hepatitis E manifestation. Understanding the mechanisms of pregnancy-associated severe hepatitis E, especially fulminant hepatitis E, in the future will help devise effective preventive measures against this disease. 

Genotypes 1 and 2 HEV strains are restricted to the human population, while Genotypes 3 and 4 HEV strains infect both humans and other animals with zoonotic transmission routes. Human to human transmission of HEV is considered rare; although, blood-borne transmission has been reported via blood transfusion [20,58,59,60]. A comparative study of Genotype 3 and 4 HEV-infected individuals in Japan revealed that Genotype 4 HEV is associated with a higher level of alanine aminotransferase (ALT), higher prevalence of clinical infection, higher level of total bilirubin, higher level of viremia, more frequent fulminant hepatitis development, and overall a more aggressive hepatitis [48]. The mechanisms of cross-species HEV infection remain poorly understood. Identification of both the viral genetic elements and host factors that are important for cross-species HEV infection will be the key for devising strategies to prevent and control zoonotic HEV infections. 

### 3.2. HEV Infection in Animals

Natural and experimental HEV infections in swine (Genotypes 3 and 4) result in a subclinical course of infection with only mild microscopic lesions in the liver and associated lymph nodes [61,62]. Viremia lasts 1–2 weeks with fecal virus shedding lasting 3–7 weeks [7,61,62]. HEV infection in swine is age-dependent with up to 86% of the pigs infected by 18 weeks of age [63]. Additional studies from the United Kingdom, Spain, and Japan further demonstrated that the highest fecal virus shedding occurred by 10–12 weeks, 13–16 weeks, and 1–3 months of age, respectively [64,65,66]. Seroconversion to HEV antibodies in swine occurs following the typical waning in maternal antibody levels around 8–10 weeks of age first with IgM anti-HEV antibodies peaking in conjunction with fecal viral shedding followed by IgG anti-HEV antibodies peaking in conjunction with clearance of virus from the feces [20,64,65,66].Transmission between swine is fecal-oral with large amounts of infectious HEV being shed in the feces, and direct contact between animals, with other animals’ excreta, and with potentially contaminated water sources in swine facilities contributes to transmission within a herd [7,67,68,69,70]. Although HEV infection in pigs does not pose a major economical concern in swine production, the risk of zoonotic transmission to humans is an important public health concern. Therefore, development of an effective vaccine to immunize susceptible swine herds in the future will minimize the risk of zoonotic infection and improve pork safety. 

Avian HEV Genotypes 1–3 carry a slightly different course of infection with a high level of subclinical infection in flocks and mortality rates up to 0.3–1.0% [36,71,72]. Clinical signs may include egg drop in some flocks up to 20%, enlargement of the liver and spleen, and acute death of affected birds [73]. Post-mortem evaluations show enlarged, hemorrhagic, and focally necrotic livers, inflammatory cellular infiltrations in the liver tissue, serosanguinous abdominal fluid, and regressing ovaries in some affected birds [73,74]. It appears that avian HEV does not infect humans, and thus is not a concern for food and environmental safety. Nevertheless, more studies are needed to fully assess the potential of avian HEV cross-species infection. 

## 4. Epidemiology of HEV Infection

HEV is considered hyperendemic in many developing countries such as India, Bangladesh, Egypt, Mexico, and China. Hyperendemic countries carry an HEV prevalence of 25% of all non-A, non-B, acute hepatitis cases or have experienced a major waterborne outbreak of hepatitis E according to the Centers for Disease Control and Prevention [75]. HEV is considered endemic where there is a prevalence of less than 25% of all reported non-A, non-B acute hepatitis [75]. Endemic countries include much of Western Europe, the United States, New Zealand, many countries in South America, much of Asia, and the Middle East [75,76,77]. Trends throughout the World point to continued high anti-HEV seroprevalence and HEV infection likely due to increases in interest, awareness and surveillance efforts as well as increased spread among known animal reservoirs and hosts [20,75,76,77,78,79,80,81]. Seroprevalence reports vary dramatically from country to country and study to study with some studies reporting overall declines in seroprevalence over time, while other yield continued high levels of seroprevalence [80,82,83]. Prevalence of anti-HEV IgG tends to increase with age, especially in men [80,84,85,86,87]. Humans and other animals excrete a considerable amount of virus early in the acute phase of HEV infection and likely contribute to maintain the cycle of endemicity [76]. The lack of a standardized serological assay further complicated the interpretation of the sero-epidemiological data. Therefore, development of a FDA-approved diagnostic assay for HEV should be a priority in the future. 

## 5. Environmental Contamination and Waterborne Transmission

### 5.1. HEV Transmission from Sewage and Animal Manure Run-off

HEV is typically transmitted via fecal-oral route within an animal species, from animals to humans in infectious body fluids, and from contaminated food or water sources to humans and other animals. Inadequate disposal and treatment of sewage and contamination of drinking and irrigation water lead to the many epidemics in developing countries [2,88,89]. Increased rates of human HEV infection in Turkey and certain countries in Southeast Asia are associated with utilizing untreated river water for everyday tasks such as bathing, drinking, and disposal of waste products [90,91,92,93]. Environmental catastrophes and annual flooding are also associated with elevated HEV attack rates especially in regions where river, pond, or well water use is prevalent [10,92,93,94]. In both industrialized and developing countries, raw sewage water has been shown to contain infectious HEV strains that are closely related to the strains circulating in humans (Genotypes 1 and 2) and other animals (Genotypes 3 and 4) [95,96,97,98,99]. In The Netherlands, Genotype 3 HEV RNA was detected in river water which likely originated from sewage [100]. Run-offs from animal facilities such as hog operations have been implicated in human HEV infections with the detection of infectious Genotype 3 HEV in the animal manure and wastewater [100,101].

Professionals working in close proximity to swine, swine manure, or sewage may become infected with HEV during occupational activities [70,100,102,103,104]. For example, swine workers in Valencia, Spain were found to be 5.4 times more likely to be positive for anti-HEV IgG than those not exposed to swine [104]. Utilizing a Bayesian model to account for imperfections in sero-assays leading to differences in the interpretation of serology results, Bouwknegt *et al*. [103] found that approximately 11% of swine veterinarians, 6% of non-swine veterinarians, and 2% of the general population were positive for anti-HEV antibodies. Variation in assays, validity of serologic tests for determining HEV prevalence, the lack of standardized diagnostic tools, the potential for multiple routes of transmission, and incompletely understood transmission routes particularly in small defined populations lead to difficulty in assessing the exact risk factors for HEV infection [105,106]. For example, Vulcano *et al*. [107] identified male housekeepers and specific pig breeders as carrying a higher prevalence of IgG anti-HEV seropositivity than previously identified in Italy and found a 5.5% seropositivity in subjects from Rieti in comparison to 2.5% from Rome, despite an overall lack of association with swine contact. In addition, pig farmers and the general population in Sweden were found to have 13% and 9% seropositivity respectively, which was higher than previously reported for populations in Europe (1–9%) and contributes to uncertainty in our current knowledge of transmission routes and risk factors for HEV infection [108]. Again, standardized serological and molecular diagnostic tests are in critical need for the study of HEV transmission and prevalence. During natural contact routes of transmission, HEV RNA is also detectable in the urine of infected swine, which likely contributes to the ease of spread in confined swine operations and may pose as an alternate route of exposure for humans [109]. Contaminated water and sewage may serve as sources for HEV infection in both humans and other animals. Current research indicates the potential for transmission through these sources; however, further analysis of these sources in regards to all genotypes of HEV will better assess the overall public health risk.

### 5.2. Surface Water Contamination and Transmission of HEV

Surface water is easily contaminated by stable fecal-shed viruses such as HEV and acts as a public health hazard [110]. The quality of surface water directly affects populations utilizing the source since drinking water, and intensive farming practices lead to higher detection rates of viruses within these sources [110,111]. In Canada, HEV Genotype 3 detected from field-grown strawberries shared 99% nucleotide sequence identity with local swine HEV strains [112,113]. In Slovenia, Genotype 3 HEV was recovered from surface waters as well as from 20% of fecal samples in local pig farms [114]. Typical irrigation practices allow HEV and other enteric and hepatic viruses to impact surface water quality and elevate the potential for human exposure to pathogens [115,116]. Contaminated produce may serve as a source for autochthonous HEV cases in non-endemic regions [112,117]. In all cases of HEV detection in water or produce, the contamination levels were not assessed for further infectivity of humans or animals. The ability to recover infectious virus both from the local pig farms, the surface waters, and from produce receiving contaminated water would indicate that the virus is stable enough to be transmitted in these sources. Therefore, further infectivity studies should be done to assess the ability to transmit and cause infection especially in cases where the virus contamination levels are low.

### 5.3. Coastal Water Contamination and Transmission of HEV

Coastal waters may also be contaminated by HEV leading to accumulation of the virus in the digestive tissues of shellfish, which poses a risk of human infection through ingestion. Most often, mussels, cockles, and oysters are eaten raw or slightly cooked, and HEV is stable in both alkaline and acidic environments, frozen for more than 10 years, and remains infectious at up to 60 °C, suggesting that a raw, rare-cooked, or slightly steamed contaminated seafood may transmit HEV to consumers [118,119]. Shellfish have been implicated in an outbreak of HEV occurring aboard a cruise ship in European waters and HEV has been identified in commercial mussels obtained from three European countries (Finland, Greece, and Spain) [120,121]. In Scotland, 92% of bivalve mussels collected were tested positive for HEV RNA with the viral sequences clustering with Genotype 3 human and swine HEV [122]. Case reports of hepatitis E in England, Italy, and France reveal shellfish consumption as a common source risk factor for HEV infection [79,123,124]. In addition, Genotype 3 swine HEV has been detected in shellfish in Korea and Japan [125,126,127]. Travelers to hyperendemic and endemic regions of the world are at an increased risk of acquiring HEV infection from contaminated water and seafood, but industrialized countries are not exempt [77].

## 6. Foodborne Transmission and Food Safety

The meat products from HEV-infected reservoir animal species are capable of transmitting HEV to humans and are a public health concern [75,76,88]. HEV primarily replicates in the liver of infected animals; however, extra-hepatic sites of HEV replication have also been demonstrated in the gastrointestinal tissues, mesenteric and hepatic lymph nodes, and spleen [20]. In addition to the liver tissues, HEV RNA has been detected from the stomach, kidney, salivary glands, tonsils, lungs, and multiple muscle masses of pigs and chickens when inoculated intravenously [128,129,130]. 

Consumption of undercooked or raw organs or tissues from infected swine has been linked to numerous cases of hepatitis E worldwide. For example, three cases of hepatitis E in Japan were associated with the consumption of undercooked or raw pork presumably from the same barbeque restaurant [131]. Nine of ten clinical cases of hepatitis E from 2001 to 2002 had a history of consuming undercooked pork 2–8 weeks before the onset of clinical signs and 1.9% of pig livers tested from local groceries in Hokkaido, Japan were positive for Genotype 3 or 4 HEV RNA [13]. Consumption of pig liver or intestines is considered as a risk factor for HEV infection [131]. Cases of hepatitis E in Japan were also linked to the consumption of contaminated wild boar meat [132,133,134,135]. Wild boar populations in Italy and South-eastern France had detectable levels of HEV RNA in 2.5% of liver samples and 25% of bile samples, respectively [136,137]. Boar meat consumption was positively associated with HEV infection in a case-control study in Germany [138]. Cases of acute hepatitis E associated with Genotype 4 HEV have been confirmed in South Korea, presumably due to the consumption of raw wild boar bile juice [139]. Human patients with acute HEV infections in France were linked to the consumption of figatellu sausage (Corsican raw pig liver dish). The HEV sequences recovered from the figatellu products in local grocery stores were essentially indistinguishable from the viral sequences recovered from the human patients, thus providing compelling evidence for foodborne HEV transmission [11,140]. The HEV present in the pig liver sausage from manufacturers in France was shown to be infectious utilizing a 3D HEV cell-culture system [141]. Commercial pig livers tested in the United States, Germany, and The Netherlands also carried detectable levels of HEV RNA in 11%, 4%, and 6.5% of the samples tested, respectively [142,143,144]. At slaughterhouses in Bavaria, Germany, 68.6% of the serum samples and 67.6% of meat juice samples were tested seropositive for HEV antibody, indicating animal exposures to HEV prior to slaughter [145]. In Italy, an overall 87% anti-HEV seropositivity was detected in slaughterhouse swine and 64.6% were positive for HEV RNA indicating both a high level of exposure to HEV and a similarly high level of active virus infection at the time of slaughter [146]. Similar investigations of pork production chains in the Czech Republic, Spain, and the United Kingdom revealed detectable, infectious HEV at both processing locations and point of sale [147]. Genotype 4 HEV has also been identified in a small percentage of pig livers collected from markets in India and carry a 90–91% nucleotide sequence identity with the local swine HEV isolates [148]. Other reports identify Indian strains of Genotype 4 swine HEV as genetically distinct from Genotype 1 human HEV strains circulating in the region further convoluting the route of transmission [149]. Human consumption of Genotype 4 HEV-contaminated pork livers leading to disease has not yet been reported in India, which may be due to differing culinary habits [11,140]. It is likely that the Genotype 4 swine HEV in India does cause sporadic cases of acute hepatitis E in humans through zoonotic infection, although such rare and sporadic cases of Genotype 4 hepatitis E may be masked by the more prevalent and explosive form of Genotype 1 hepatitis E in India.

In addition to pork, game meats such as deer have also been implicated as sources for HEV transmission to humans following the detection of near identical HEV sequences from leftover Sika deer meat and four hepatitis E patients in Japan who previously consumed the deer meat as sushi [14,20,150]. A locally caught wild deer carried a nearly identical HEV isolate that was later confirmed in local wild boar populations in Japan as well [150]. Sashimi style deer meat is usually consumed in Japan where a case-control study attributed raw deer meat as a risk factor for anti-HEV seropositivity after identifying a positive association between deer meat consumption and a previous case of hepatitis E [14,151]. Elevated risks indicate that within this defined case-control population, those who consumed raw deer meat were more likely to be positive for HEV antibodies indicating exposure to the virus, while those who did not consume the deer meat had a lower level of exposure based on seropositivity [14,151]. Consumption of game meats including wild boar, deer, and hare was independently associated with HEV infection in organ transplant recipients in France with an odds ratio of 2.32 [152]. Combined, these studies clearly identify wild and domestic pork products and game meats as sources for human HEV infection and implicate foodborne transmission as a common route for HEV infection. 

## 7. Known and Potential Animal Reservoirs

A number of animals are known to serve as the natural hosts and reservoirs for HEV. HEV has been genetically identified from rat, wild boar, domestic swine, mongoose, rabbits, chickens, ferrets, cutthroat trout, bats, and deer [17,18,19,23,25,26,34,61,139,153]. Anti-HEV antibodies have been detected in a number of other animal species including cattle, sheep, and goats with the potential to carry novel strains of HEV [1,154]. With the advance of modern molecular biology techniques such as metagenomics and pyrosequencing, it is expected that the host range of HEV will expand and novel strains of HEV will be identified from other animal species in the near future. 

### 7.1. HEV in Avian Species

Avian HEV was identified as such in 2001 from chickens with Hepatitis-Splenomegaly (HS) syndrome in the United States [34]. Likewise, Big Liver and Spleen Disease virus (BLSV) in Australia presented similarly with an approximately 80% nucleotide sequence identity to avian HEV [34,73]. These two previously identified syndromes (HS and BLS) are assumed to be caused by variant strains of the same virus, avian HEV, which now encompasses three distinct, but related genotypes worldwide [24,73,155,156]. In the United States, an estimated 71% of chicken flocks and 30% of individual chickens are positive for avian HEV [36]. Avian HEV infection in chickens is age-dependent with 17% of seropositive chickens under 18 weeks of age and 36% of seropositive adult chickens [36,157]. Avian HEV has been shown to cross species barriers and infect turkeys [71]. It is currently unknown, however, whether avian HEV is capable of transmission to humans or other mammalian species; although, rhesus monkeys and mice are not susceptible to infection by avian HEV under experimental conditions [1,73].

### 7.2. HEV in Domestic and Wild Swine Species

Since its discovery in domestic swine in the United States in 1997, swine HEV strains have been identified worldwide in both domestic and wild swine with widely variable prevalence [11,61]. Studies of prevalence across Japan revealed that anti-HEV antibody is present in 93% of all domestic swine farms tested and that all swine HEV isolates belong to either Genotype 3 or 4 [24,48,158,159]. Prevalence of anti-HEV antibodies in wild boars in Japan is also widely variable ranging from 4.5% to 34.3% based on geographic regions with Genotype 3 or 4 HEV RNA detection rates ranging from 1.1% to 13.3% [48]. In The Netherlands, domestic swine farms carried a prevalence of 55% for HEV RNA in the feces, while 86.2% and 47.1% of 18 week-old pigs in Canada shed HEV virus in feces and serum, respectively, with a declination as the pigs aged [63,160]. In Spain, the prevalence of anti-HEV antibodies on commercial swine farms reached 98%, while the anti-HEV prevalence in New Zealand, Laos and Brazil is 90%, 46% and 81%, respectively [20,161,162,163,164]. The anti-HEV seropositivity in wild boars varied from 17–50.3% with HEV RNA detected in up to 25% of samples in Germany, Italy, Spain, Australia, and Hungary [15,136,137,165,166,167]. In the United States, swine HEV infection in pig farms is also widespread, and the majority of pigs became seropositive to HEV antibodies at approximately 3 months of age [61]. It appears that Genotype 3 or 4 HEV infection in pigs is widespread in the pig population worldwide, thus raising a concern for zoonotic infection and pork safety.

### 7.3. HEV in Deer

Deer have been implicated both acting as animal reservoirs for HEV and acting as vehicles for human infection [12,14,20,150]. The Sika and Yezo deer in Japan carried a 3% and 35% anti-HEV seroprevalence respectively, with a positive association with HEV infection in humans and nearly identical nucleotide sequence identity with HEV strains from local wild boars [7,14,150,168]. In Hungary, the European roe deer was implicated as a reservoir species for HEV, and in The Netherlands 5% of red deer were also found positive for antibodies to HEV [165,167,169]. White-tailed deer in Northern Mexico carried a 62.7% anti-HEV seropositivity [170]. Increasing management of deer including feeding, watering, movement of groups, and fencing for hunting purposes in Mexico offers the ability for pathogens such as HEV to transfer between groups of deer and humans readily and may serve to disseminate pathogens to animals within the United States [170]. Sharing of habitats between wild boar and deer may play a role in the ability to harbor and transmit HEV to humans. However, without additional direct evidence of transmission within the deer species, it is difficult to determine whether deer acts as incidental or natural hosts to HEV infection [1,20,76].

### 7.4. HEV in Ruminants

Ruminant (cattle, sheep and goat) strains of HEV have yet to be uncovered; however, multiple studies of anti-HEV seroprevalence indicated the possibility of their existence [7,165]. In Egypt, 11% of cows, 14% of buffalo, 4.4% of sheep, and 9.4% of goats were tested positive for HEV antibodies [171]. Approximately 4.4–6.9% of cows and 0% of goats in India, 1.4% of cows and 0% of sheep and goats in Brazil were reportedly tested positive for anti-HEV antibodies [172,173]. Reports of anti-HEV seropositivity from China varied drastically from 6–93% of cattle and 10–12% of sheep [174,175,176,177]. A short sequence (189 bp) of a Genotype 4 HEV has been reportedly identified in bovid species, although independent confirmation of this unsubstantiated report is still lacking [1,7,76].

Despite the abundant serological evidence for an HEV-related agent in ruminants, definitive genetic identification of HEV from ruminants is still lacking. It is possible that the strain carried by ruminants is very divergent genetically from the known HEV strains thus leading to failure to genetically identify the virus based upon current techniques. The serological data from ruminants is based upon cross-reaction of the ruminant serum samples with known HEV proteins such as ORF2 [7,165,171,172]. The validity of such serological data has been questioned due to the fact that the assays may not be specific, they do not identify the actual virus, and they may allow cross-reactivity with non-viral proteins that share a certain level of sequence homology. Research in this area must continue to better address these concerns and confirm the source of anti-HEV seropositivity in ruminants. Given the wide use of cattle, sheep, and goats in the human food chain, the genetic identification of these ruminant strains of HEV would be of a potential public health concern.

### 7.5. HEV in Rats

The rat strain of HEV was identified in wild Norway rats from Hamburg, Germany with 59.9% and 49.9% nucleotide sequence identity with known human and avian HEV strains, respectively [18]. Rats in the United States, Germany, Indonesia, China, and Japan are also tested seropositive for HEV antibodies in several studies with variable prevalence [18,178,179,180,181]. Overall, 44% of rats in Louisiana, 77% in Maryland, 90% in Hawaii, 59.7% of rats of the genus *Rattus* from across the United States, 32% of Norway rats in Japan, and 13% of black rats in Japan were tested positive for antibodies to HEV [178,179,182,183]. Most recently in China, 23.3% of rats were positive for anti-HEV IgG with the highest prevalence of 45.3% from rats caught at garbage dump sites [180]. In Indonesia, 18.1% of rats were tested positive for anti-HEV antibodies and 14.7% positive for HEV RNA [181]. Recently, Genotype 3 rat HEV strains have been genetically detected from wild rats in the United States, suggesting the potential for zoonotic transmission and the genetic variability of rat HEV [1,182]. Further studies are warranted to independently confirm the existence of Genotype 3 HEV in rats, especially since, under experimental conditions, laboratory rats are not susceptible to experimental infection by Genotype 3 HEV [184]. 

### 7.6. HEV in Rabbits

Rabbits may serve as reservoir hosts for HEV transmission to humans given the genetic identification of zoonotic Genotype 3 strains of HEV from rabbits in China, the United States, and France [17,153,174,185]. Rabbits are susceptible to experimental infection by Genotype 4 human HEV, and the infected rabbits developed viremia, seroconversion to anti-HEV, and fecal virus shedding [153,185]. The rabbit HEV is genetically and antigenically closely related to other mammalian HEV. The capsid protein of the Genotype 3 rabbit strain of HEV was capable of cross-reacting with antibodies from other strains of HEV including rat, swine, human, and chicken [1,185,186]. The prevalence of HEV antibodies in farmed rabbits is reportedly 57% in the Gansu province in China, 54.6% in Beijing, China, and 36.5% in two rabbit farms in Virginia, USA, while HEV RNA has been identified in 7.5%, 7.0%, 16.5%, and 15.3% of the rabbits, respectively [17,153,174]. In France, HEV RNA was also identified from 7.0% of farmed rabbits, while 23.0% of wild rabbits were also positive for HEV RNA [185]. It appears that rabbits could be an important reservoir for HEV infection in humans, and in-depth studies of its ability to infect across species barriers and associated zoonotic risks in the future are needed.

### 7.7. HEV in Other Species

Other known animal strains of HEV genetically identified thus far include mongoose, ferret, bat, and fish [1,23,25,26,187,188]. Wild mongoose in Okinawa, Japan carried Genotype 3 HEV strains and the prevalence of anti-HEV seropositivity varied from 8% to21% [187,188]. In The Netherlands, ferrets carried a strain of HEV that shared a 72.3% nucleotide sequence identity with that of the rat HEV [23]. The cutthroat trout in the United States also carried a unique strain of HEV with only 13% to 27% sequence identity with known mammalian and avian HEV strains [26]. The zoonotic potentials of these novel animal strains of HEV are not altogether understood, but the ever-expanding host range and high levels of anti-HEV seropositivity among mammalian species suggests transmission is common and thus may pose a potential public health concern.

## 8. Animal Handling and Zoonotic Transmission

Contact exposure to infected animals leads to an elevated risk for HEV transmission in humans. Swine veterinarians in the United States were shown to have a 27% seropositivity to Genotype 3 swine HEV in comparison to 16% of the normal blood donors [189]. Individuals from states in which swine production plays a key role were more likely to be seropositive to HEV than other non-major swine states [189]. Incidents such as needle sticks while working with swine were found to be1.9 times more likely positive for HEV antibodies in swine veterinarians [189]. Pig handlers such as veterinarians, breeders, and farmers in China, Thailand, The Netherlands, Sweden, Moldova, and the United States were also more likely seropositive to swine HEV [103,108,190,191,192]. In Sweden, 13% of pig breeders were positive for antibodies to HEV [190]. In The Netherlands, 11% of swine veterinarians were positive in comparison to 6% of non-swine veterinarians and 2% of the general population [108]. In North Carolina, swine handlers carried a 4.5 times higher rate of seropositivity in comparison to non-swine workers [191]. In Moldova, 51% of swine farmers were positive in comparison to 25% of non-swine occupations [103]. Taken together, swine are a major reservoir for HEV and occupational contact with infected swine is a risk factor for zoonotic HEV transmission in humans.

Contact with swine is the most widely recognized route for occupational exposure to HEV; however, the multitude of novel strains of HEV in wildlife and other domestic animal species suggest additional mechanisms of transmission. For example, field workers at the Iowa Department of Natural Resources who work with a variety of wildlife species had a higher prevalence for HEV antibodies in comparison to normal blood donors [193]. While exposure to HEV, identified by the presence of anti-HEV antibodies in these populations does not in itself indicate a disease, it does identify a route of transmission and exposure that should be further assessed and acknowledged as a preventive measure against this important disease. Examination of these additional mechanisms is vital to understanding the full-spectrum of public health risk associated with HEV infection.

## 9. Conclusions

The zoonotic risk of HEV is well established; however, the ever-expanding host range and identification of new animal reservoir species poses a significant public health concern. Seroprevalence in human and other animal species varies drastically between studies and countries with no clear understanding of the overall problem, and this is largely due to the lack of an established FDA-approved serological diagnostic assay. Numerous animal species were tested seropositive for IgG anti-HEV, although HEV was not genetically identified from all seropositive animal species. Detection of HEV in sewage, water sources, coastal and surface waters, and produce poses environmental safety concerns even in industrialized countries where waterborne origins of human hepatitis E cases were previously considered rare. Foodborne cases of hepatitis E in humans are increasingly common and likely underestimated in the medical community. Sporadic and cluster cases of hepatitis E occur after consumption of undercooked or raw animal meats. Prevention of foodborne HEV transmission relies on avoiding consumption of undercooked animal meats especially when immunocompromised, following good hygiene practices, and being aware of increased risks when traveling to endemic or hyperendemic regions of the world. Despite the clear risk, prevention strategies are currently minimally implemented. A vaccine against HEV has recently become available in China but not in other countries. Surveillance, vaccination, de-contamination of sewage and water sources, and public education will help prevent current and future endemics or epidemics lowering the human burden. The development of a vaccine against the zoonotic swine HEV would reduce foodborne and swine contact cases in humans as well as diminish the spread of the virus between animal species. Control of animal waste, run-off, and decontaminated sewage is key to limiting the spread of HEV to coastal and surface waters and in turn reducing concomitant contamination of shellfish. 
